# Histone H3 mutations—a special role for H3.3 in tumorigenesis?

**DOI:** 10.1007/s00412-015-0510-4

**Published:** 2015-03-14

**Authors:** Satish Kallappagoudar, Rajesh K. Yadav, Brandon R. Lowe, Janet F. Partridge

**Affiliations:** Department of Pathology, St. Jude Children’s Research Hospital, 262 Danny Thomas Place, Memphis, TN 38105 USA

## Abstract

Brain tumors are the most common solid tumors in children. Pediatric high-grade glioma (HGG) accounts for ∼8–12 % of these brain tumors and is a devastating disease as 70–90 % of patients die within 2 years of diagnosis. The failure to advance therapy for these children over the last 30 years is largely due to limited knowledge of the molecular basis for these tumors and a lack of disease models. Recently, sequencing of tumor cells revealed that histone H3 is frequently mutated in pediatric HGG, with up to 78 % of diffuse intrinsic pontine gliomas (DIPGs) carrying K27M and 36 % of non-brainstem gliomas carrying either K27M or G34R/V mutations. Although mutations in many chromatin modifiers have been identified in cancer, this was the first demonstration that histone mutations may be drivers of disease. Subsequent studies have identified high-frequency mutation of histone H3 to K36M in chondroblastomas and to G34W/L in giant cell tumors of bone, which are diseases of adolescents and young adults. Interestingly, the G34 mutations, the K36M mutations, and the majority of K27M mutations occur in genes encoding the replacement histone H3.3. Here, we review the peculiar characteristics of histone H3.3 and use this information as a backdrop to highlight current thinking about how the identified mutations may contribute to disease development.

## Introduction

Chromatin is made up of nucleosomes comprising histone octamers with a stable tetrameric core of histones H3 and H4, flanked by two more labile dimers of histone, H2A and H2B. Each histone octamer is wrapped by 147 bp DNA, which facilitates the compaction of genomic DNA and regulates access to regulatory factors (Workman and Kingston 1998). Chromatin is critical for the regulation of genome stability and for transcriptional control and its importance in disease has been highlighted by the frequent identification of mutations in chromatin-modifying enzymes in cancer genomes (Plass et al. 2013; Huether et al. 2014). Intriguingly, sequencing of pediatric high-grade gliomas identified high-frequency mutations in a core histone subunit, H3 (Schwartzentruber et al. 2012; Wu et al. 2012), and subsequent studies have identified histone H3 to be mutated in virtually all cases of chondroblastoma and giant cell tumors of bone (Behjati et al. 2013), diseases of adolescents and young adults. The majority of the mutations have been identified in genes encoding histone H3.3, which serves as a replacement histone as its deposition is not coupled to DNA synthesis. Here, we review the specific characteristics of histone H3.3, the spectrum of mutations identified in tumors, and recent work directed at understanding how mutation of this protein contributes to disease.

## Histone H3.3—a variant of a core nucleosomal protein

**Fig. 1:**
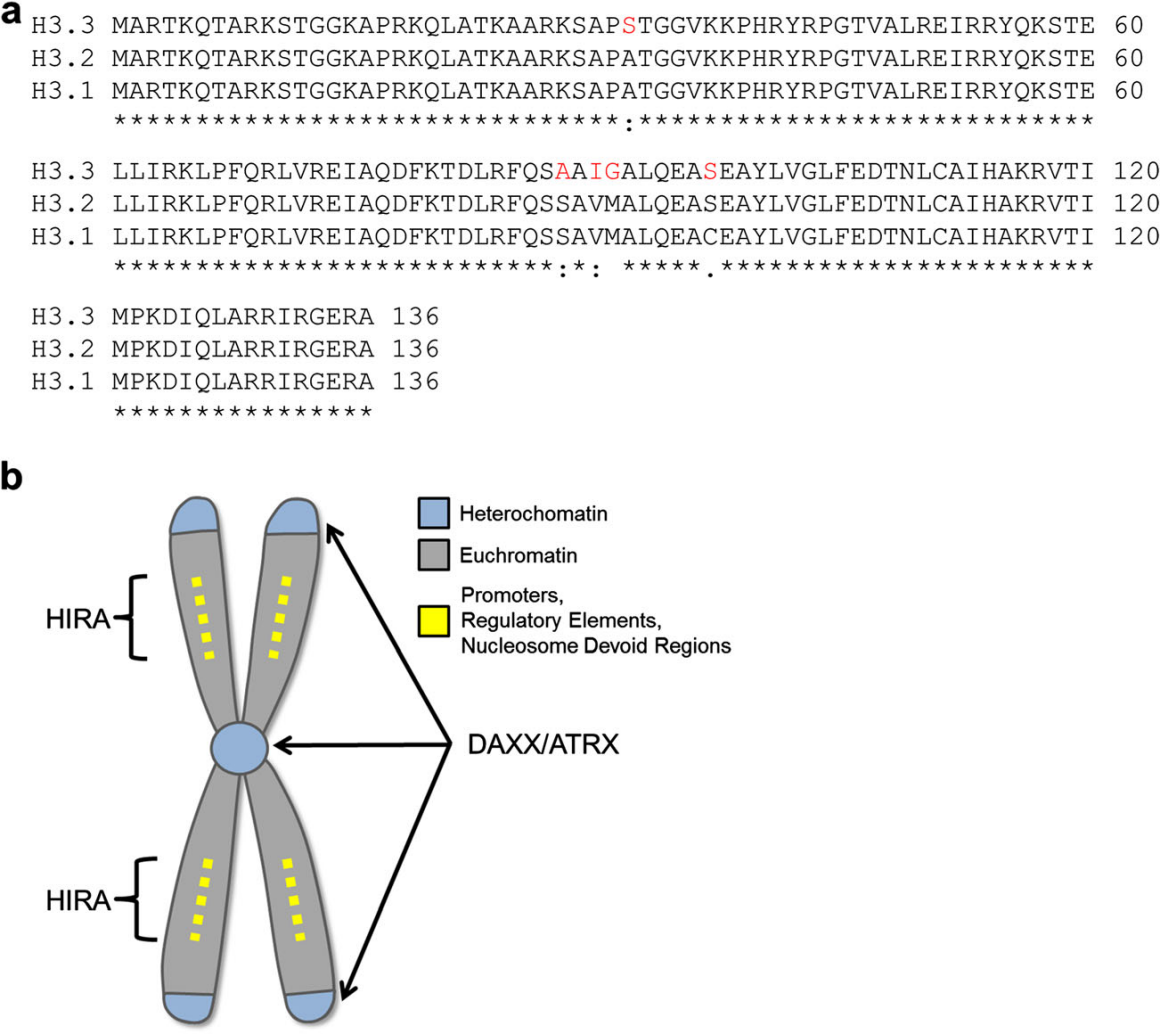
Histone H3.3 shows amino acid differences with H3.1 that promote binding to distinct chaperones. **a** Sequence alignment of human H3.3, H3.2, and H3.1, with sequence differences in H3.3 marked in *red*. **b** Cartoon of chromosome depicting regions of H3.3 incorporation into chromatin and the chaperones responsible

Several flavors of histone H3 are expressed in higher eukaryotes—including histone H3.1, H3.2, H3.3, and a centromere-specific H3 variant protein, CENP-A. Histones H3.1 and 3.2 are synthesized during S phase (Osley 1991), are incorporated de novo into newly replicated chromatin as well as during DNA repair, and are thus termed “DNA synthesis-coupled.” In contrast, the “replacement histone” H3.3 is expressed throughout the cell cycle, as well as in quiescent cells (Wu et al. 1982), and is largely deposited in a DNA synthesis-independent fashion by a distinct set of chaperones, proteins which associate with soluble histones and control the assembly (or disassembly) of nucleosomes from histones and DNA. Histone H3.1 differs from H3.2 by a single amino acid (Ser^96^ in H3.2), and H3.3 is distinguished by an additional four amino acid substitutions (Ser^31^, Ala^87^, Ile^89^, Gly^90^) (Franklin and Zweidler 1977) (Fig. 1a). These clustered amino acids that differ between H3.1 and H3.3 have been linked to the differential binding of chaperones (Tagami et al. 2004; Drane et al. 2010; Lewis et al. 2010; Wong et al. 2010), with specifically G90 of H3.3 promoting binding to DAXX (death-domain associated protein) (Elsasser et al. 2012).

The DNA synthesis-coupled histones are encoded by unusual transcripts that lack introns and polyadenylation signals: histone H3.2 is encoded by three genes (*HIST2H3A*, *HIST2H3C*, and *HIST2H3D*), whereas H3.1 is encoded by ten genes clustered on chromosome 6. The DNA synthesis-independent H3.3 is expressed from only two genes, *H3F3A* on chromosome 1 and *H3F3B* on chromosome 17. These genes produce identical proteins even though they have distinct regulatory sequences and yield distinct polyadenylated transcripts with unusually long 5′ and 3′UTRs (Wells and Kedes 1985; Wells et al. 1987).

The relative levels of H3.1 and H3.3 have been measured in several cell types and range from ∼20–50 % H3.3 and ∼20–70 % H3.1 in actively dividing cells (Hake et al. 2006). However, given the cell cycle dependence of synthesis of H3.1 and H3.2, the relative abundance of H3 variants differs substantially between tissues and during development (Gabrielli et al. 1984; Frank et al. 2003). Accordingly, post mitotic cells, such as cerebral cortical neurons, accumulate high levels of nucleosomal H3.3 (87 % of nucleosomal H3 content) as DNA synthesis-independent H3.3 deposition is ongoing while replication-coupled 3.1 and 3.2 deposition stops during gestation (Pina and Suau 1987).

The DNA synthesis-coupled and DNA synthesis-independent H3s show distinct localization patterns across the genome. H3.1 is incorporated universally in the S phase by CAF1 (chromatin assembly factor) (Gaillard et al. 1996; Tagami et al. 2004; Ray-Gallet et al. 2011) which is recruited to newly replicated DNA by PCNA (proliferating cell nuclear antigen) (Shibahara and Stillman 1999). DNA synthesis-independent H3.3 deposition occurs on DNA sequences that are transiently nucleosome-free, for example, during transcription and DNA repair, and to replace nucleosomes evicted by chromatin remodelers (Filipescu et al. 2013). H3.3 is also enriched in heterochromatic subtelomeric and pericentromeric regions. This seemingly contradictory pattern of H3.3 localization is due to the different chaperones that bind H3.3 (Fig. 1b). Deposition of H3.3 into heterochromatic loci is targeted by DAXX (Lewis et al. 2010; Drane et al. 2010) in complex with the SNF2-like remodeler ATRX (α-thalassaemia/mental retardation syndrome X-linked) whereas the HIRA chaperone inserts H3.3 into genic loci (Goldberg et al. 2010). H3.3 accrues at actively transcribed regions (Ahmad and Henikoff 2002; Schwartz and Ahmad 2005; Chow et al. 2005; Jin and Felsenfeld 2006) and can serve as a marker of regions of high transcriptional activity because it is preferentially deposited (over H3.1 and H3.2) in transcribed regions, and its histone tail is highly enriched for covalent modifications or “marks” associated with transcriptionally active chromatin, such as tri-methylation of lysine 4 (K4me3) (McKittrick et al. 2004; Hake et al. 2006; Loyola et al. 2006).

There is also conjecture over whether H3.3 is not just a marker for active regions, but whether it contributes to the transcriptional activity of loci enriched for this histone (Huang and Zhu 2014). This is in part due to its enrichment at regulatory regions such as promoters and enhancers, where it is thought to contribute to chromatin plasticity, or the openness of chromatin with enrichment for modifications associated with active chromatin and evidence of enhanced rates of nucleosome turnover and dynamic association of chromatin-associated proteins in these domains. Importantly, H3.3 is incorporated into Polycomb response elements (PREs) in *Drosophila* (Mito et al. 2007), regions of the genome that play critical roles in controlling gene expression during development, and is similarly enriched at promoter regions of developmentally regulated genes in embryonic stem cells (ESCs) by HIRA (Goldberg et al. 2010; Kraushaar et al. 2013; Huang et al. 2013). Cells depleted for H3.3 show decreased levels of nucleosome turnover at sites of H3.3 incorporation (Kraushaar et al. 2013; Huang et al. 2013; Banaszynski et al. 2013), correlating with defective expression of developmentally regulated genes on ESC differentiation (Banaszynski et al. 2013).

H3.3 plays important roles in many developmental contexts (reviewed in Filipescu et al. 2013). H3.3 plays critical roles in stem cells, during fertilization and reproduction and during reprogramming of genomes following fertilization or somatic cell nuclear transfer (van der Heijden et al. 2005; Loppin et al. 2005; Torres-Padilla et al. 2006; Hodl and Basler 2009; Sakai et al. 2009; Santenard et al. 2010; Banaszynski et al. 2013; Wen et al. 2014a; Wen et al. 2014b). H3.3 is also preferentially cleaved at residue 21 during senescence to lock in the senescent cell fate, presumably by removal of “active” K4me3 marks (Duarte et al. 2014).

Cells in which H3.3 has been knocked down are also susceptible to DNA damage. This can be explained by the requirement for H3.3 in a “gap-filling” mechanism to ensure nucleosome replacement in transcriptionally active areas (Ray-Gallet et al. 2011). H3.3 is incorporated at sites of UV damage, it protects against sensitivity to UV light and is required to maintain replication fork progression after UV damage (Frey et al. 2014). H3.3 may also be important for the restart of transcription following DNA damage since knockdown of the H3.3 chaperone HIRA resulted in an impaired recovery of RNA synthesis after UVC damage (Adam et al. 2013). Thus, H3.3 plays many diverse roles in chromatin regulation and is the subject of active study. Interest in H3.3 has increased even more with the surprising finding that the core histone H3 protein, and in particular, H3.3, is affected by specific mutations in several tumors.

## Mutational spectrum of histone mutant tumors

**Fig. 2:**
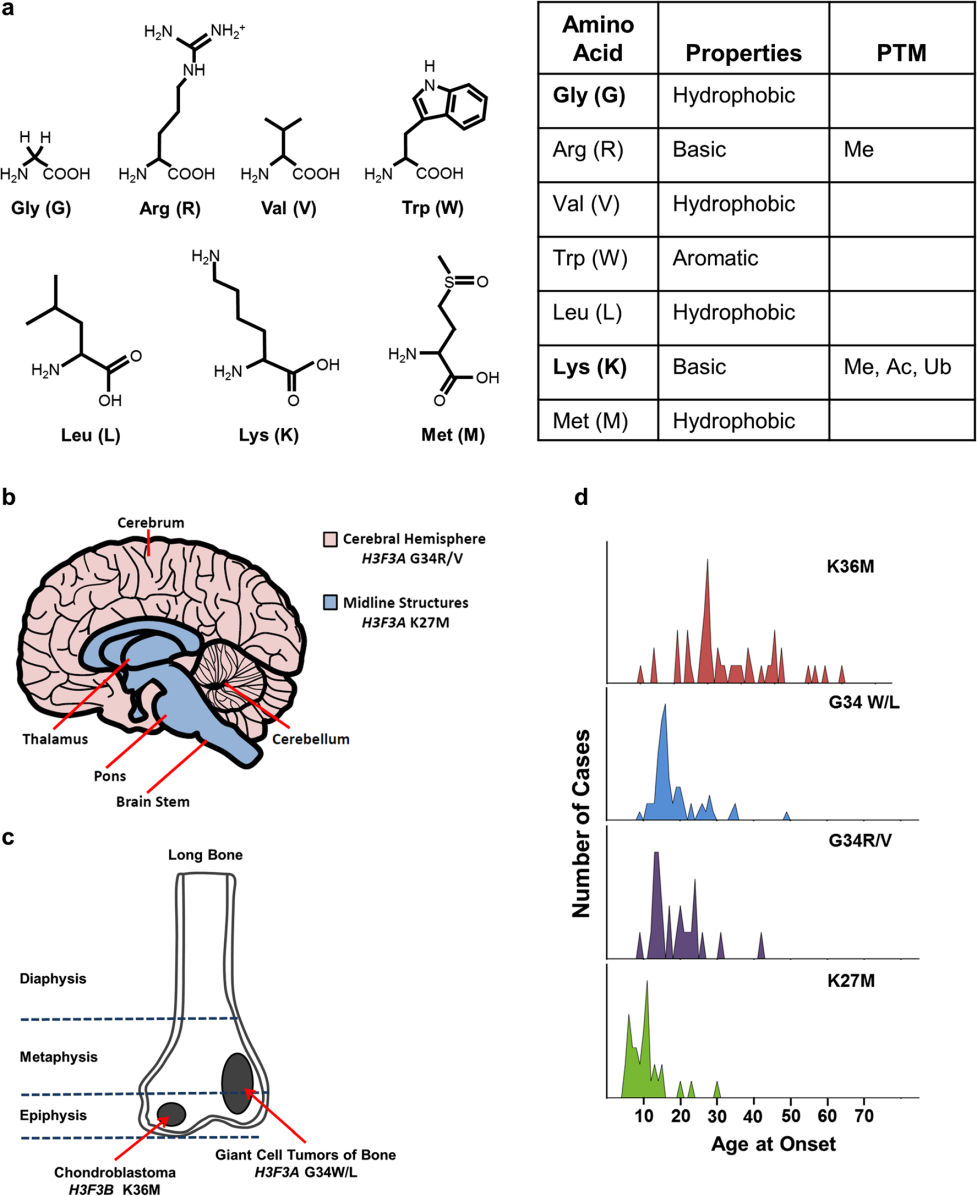
Specific histone H3 mutants arise in distinct regions of the brain or in different skeletal tissues and show variable age of presentation. **a** The amino acids that substitute glycine at amino acid 34 or lysine at amino acids 27 and 36 of histone H3, their properties, and possible posttranslational modifications. **b** Cartoon depicting the different anatomical location of brain tumors bearing K27M mutant H3.1 or H3.3 and G34R or G34V mutant H3.3. K27M mutants are predominantly found in midline structures (including the thalamus, pons, and brainstem), whereas the G34 mutant tumors are most commonly located in the cerebral hemispheres (Sturm et al. 2012; Bjerke et al. 2013). **c** Cartoon illustrating the distribution of different histone H3 mutants in chondroblastomas and giant cell tumors of bone (Behjati et al. 2013). **d** Graphical representation of span of age of presentation for histone H3 mutant tumors (Schwartzentruber et al. 2012; Sturm et al. 2012; Behjati et al. 2013)

Histone H3 has recently been found to be mutated at high frequency in several specific cancer types including pediatric high-grade glioblastoma (HGG), chondroblastoma, and giant cell tumors of the bone (Fig. 2). The identified missense mutations affect only three specific amino acids in the N-terminal tail of histone H3, a region of extensive posttranslational modification, and were found predominantly in the genes encoding H3.3, *H3F3A*, and *H3F3B*, and to a lesser extent in H3.1 genes, *HIST1H3B* and *HIST1H3C*. Such specificity and frequency of mutation allow these mutations to be defined as “Driver mutations” for tumorigenesis (Vogelstein et al. 2013). The specific mutations of H3.1 and H3.3 were found to vary by tumor type, patient age, and location, with each tumor containing a single mutant H3 allele (Khuong-Quang et al. 2012; Sturm et al. 2012; Schwartzentruber et al. 2012; Wu et al. 2012; Gielen et al. 2013).

**Fig. 3:**
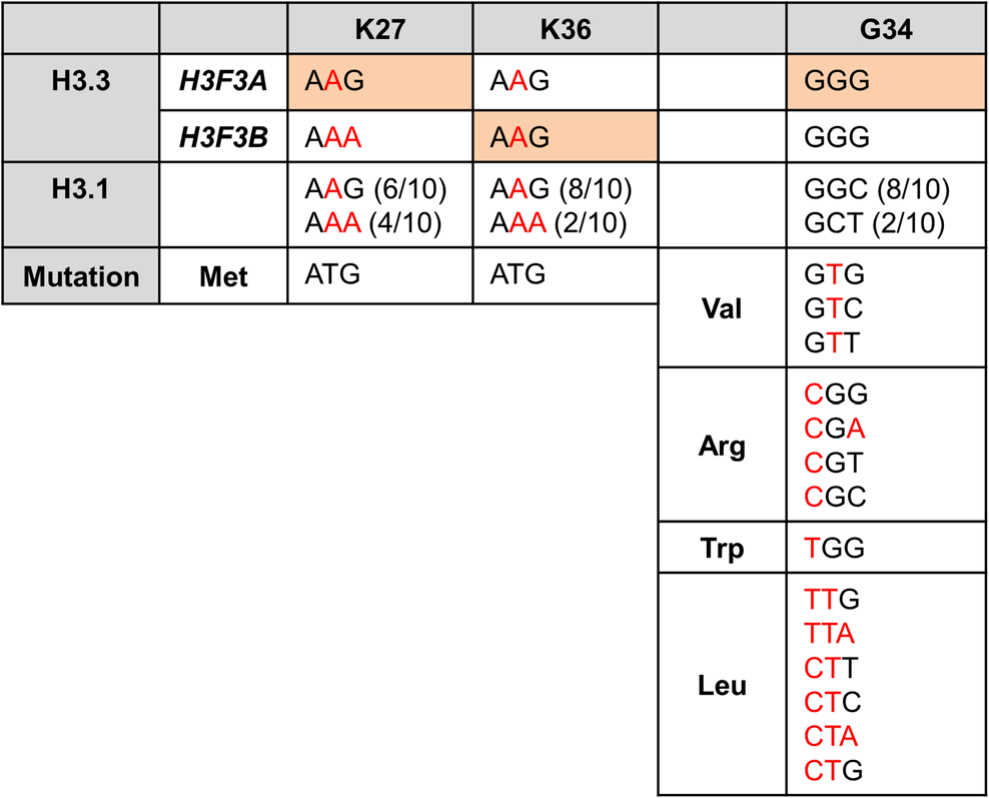
Codon usage in histone H3.3 and H3.1 genes at the sites of histone mutation. *Orange-shaded boxes* mark the genes in which mutations are prevalent for the different amino acid substitutions

Sequencing of pediatric HGG tumors identified a recurring somatic mutation of H3 lysine 27 to methionine (K27M) in ∼30 % pediatric HGG tumors (Wu et al. 2012; Schwartzentruber et al. 2012), mainly in tumors of the midline such as the thalamus, basal ganglia, and spinal cord (Sturm et al. 2012). Evidence for the mutation being somatic derives from sequencing of matched normal DNA from patients carrying histone mutant tumors, which in every case (39 patients) demonstrated the somatic nature of the mutation (Wu et al. 2012). The K27M mutation is most often found in *H3F3A* (>70 %) with a few occurrences in *HIST1H3B* (∼20 %) and *HIST1H3C* mainly in younger patients with a median age of 10–11 years (Wu et al. 2012; Schwartzentruber et al. 2012; Sturm et al. 2012; Khuong-Quang et al. 2012; Wu et al. 2014; Fontebasso et al. 2014; Buczkowicz et al. 2014). Analysis of the DNA sequences for *H3F3A* and *H3F3B* illuminates why the K27M mutation is restricted to *H3F3A*, as K27 is coded by AAG in *H3F3A* and by AAA in *H3F3B*, requiring a single mutation in *H3F3A* to generate ATG to code for methionine (Fig. 3). Why the mutation has only been found in *HIST1H3B* and *C* is less clear since six of the H3.1 genes have AAG coding for K27. This selection may be explained by differences in expression patterns of the different H3.1 encoding genes. Data from several groups indicate that diffuse intrinsic pontine glioma (DIPG), a tumor of the pons, has an even higher incidence of K27M, with >70 % of tumors sequenced containing the mutation as compared to less than 25 % of non-brainstem gliomas (Khuong-Quang et al. 2012; Schwartzentruber et al. 2012; Sturm et al. 2012; Wu et al. 2012; Gielen et al. 2013). DIPG tumors are particularly deadly, with a median age of onset of 8 years and survival rates of ∼10 % at 2 years post-diagnosis (Khuong-Quang et al. 2012). Whether this poor outcome is linked to the preponderance of the K27M mutation or to the inability to surgically resect these tumors can now be assessed using immunohistochemical staining of all HGG tissue samples with newly developed diagnostic anti-H3K27M antibodies (Venneti et al. 2014; Bechet et al. 2014).

Around 30 % of non-brainstem pediatric glioblastoma tumors bear histone H3 mutations, including thalamic K27M mutations. In cortical HGGs, ∼15 % bear a distinct mutation in histone H3.3, of glycine 34 to arginine (G34R) or, much less frequently, to valine (G34V). G34R/V containing tumors tend to be located in the cerebral hemisphere of the brain with nearly all G34R/V mutations found in *H3F3A*. G34 is coded by identical sequences in *H3F3A* and *H3F3B*, and R and V substitutions can be achieved with single point mutations. Similarly, G34R or V substitutions could be achieved by a single point mutation of any of the H3.1 genes, so other factors must determine the prevalence of *H3F3A* mutations in the tumors. G34 mutation is associated with global DNA hypomethylation, which is particularly pronounced in telomeric regions (Sturm et al. 2012). No G34R/V mutations were found in DIPG tumors, and the median age of G34 mutant tumor occurrence was older than for K27M mutant tumors (Schwartzentruber et al. 2012; Wu et al. 2012; Gielen et al. 2013).

Additional sequence analysis of DIPG tumors has highlighted the complexity of the genomic landscape, with identification of additional driver mutations that overlap or are excluded from tumors bearing mutant histones. In nearly 30 % of glioblastoma tumors, the *H3F3A* K27M mutation was observed in concert with mutations in ATRX or less commonly in DAXX (Khuong-Quang et al. 2012; Schwartzentruber et al. 2012), proteins involved in the deposition of H3.3 at regions of heterochromatin. Other studies show only a minor overlap between ATRX mutation and K27M (Fontebasso et al. 2014; Wu et al. 2014) or no mutations in ATRX in K27M tumors (Taylor et al. 2014a). Also, K27M tumors often contain mutations in the tumor suppressor protein p53, with nearly 60 % harboring the mutation (Khuong-Quang et al. 2012; Schwartzentruber et al. 2012). G34R *H3F3A* showed a significant overlap with mutations in *ATRX*/*DAXX* and *p53* with nearly 100 % of the tumors containing both mutations (Schwartzentruber et al. 2012). Other somatic mutations that appear linked to H3K27M mutation are activating mutations in *ACVR1* (activin receptor type 1) that enhance BMP (bone morphogenetic protein) signaling (Fontebasso et al. 2014; Wu et al. 2014; Buczkowicz et al. 2014; Taylor et al. 2014a). Interestingly, mutation of *ACVR1* (which occurs in ∼24 % DIPGs) was linked to the presence of *HIST1H3B* mutation (H3.1 K27M) (Buczkowicz et al. 2014; Wu et al. 2014; Fontebasso et al. 2014; Taylor et al. 2014a) and was associated with a younger age of onset of disease (Wu et al. 2014). These findings may point to a developmentally distinct cell of origin for *ACVR1*-associated tumors (Taylor et al. 2014b). Interestingly, while *ACVR1* mutations suffice to increase proliferation of immortalized normal human astrocytes (Buczkowicz et al. 2014), mutations in the identical amino acids are found in the germline of individuals with the autosomal dominant congenital childhood disorder FOP (fibrodysplasia ossificans progressiva), who have no evidence of cancer predisposition (Jones and Baker 2014; Taylor et al. 2014b). Thus, *ACVR1* mutation likely provides a selective advantage in the presence of other critical mutations, but cannot initiate tumorigenesis, as supported by the failure of *p53* null mouse astrocytes that express ACVR1 mutants to initiate tumorigenesis when implanted in the brain (Wu et al. 2014). *IDH1* mutations which are common in glioblastoma of young adults showed no overlap with *H3F3A* mutations (Sturm et al. 2012). Inactivating mutations were also identified in the histone H3 K36 trimethyltransferase, *SETD2*, in ∼15 % of pediatric HGG. *SETD2* mutations were initially thought to be restricted to cerebral hemisphere tumors and to show no overlap with *H3F3A* mutations (Fontebasso et al. 2013), but *SETD2* has been found to be mutated in a DIPG bearing a H3.1 K27M mutation (Wu et al. 2014).

Mutations in H3.3 have also been found in several types of bone tumors, with the greatest incidence in chrondroblastoma and giant cell tumors of the bone (Behjati et al. 2013). Chondroblastoma arises in children and in young adults in the cartilage of the growth plates of the long bones and is most typically benign. Sequencing of *H3F3A* and *H3F3B* in more than 70 chrondroblastomas revealed nearly 95 % of the tumors contained lysine 36 to methionine (K36M) substitutions, which mutate the target site for SETD2 and other K36 methyltransferases. Unlike the K27M mutation of glioblastoma, nearly all of the K36M mutations were found in *H3F3B* (∼90 %) rather than in *H3F3A*, which cannot be explained by differences in codon usage between *H3F3A* and *H3F3B*. Giant cell tumors of the bone also have a high frequency of H3.3 mutations, with greater than 90 % of tumors sequenced containing substitutions of G34 to either tryptophan (G34W) or, in rare cases, leucine (G34L). These frequencies can be explained by differences in gene sequence. A single base substitution suffices to mutate H3.3 G34 to W, whereas two or three mutations would be required to convert H3.1 G34 to W, and two mutations are required in any of the genes to generate a codon for leucine at position 34. In contrast to the relative genetic complexity of the pediatric high-grade gliomas, the genome of these skeletal tumors is relatively stable, with cells being diploid and wild-type for p53 (Behjati et al. 2013). This suggests that these H3.3 mutations may not only be defined as oncogenic drivers because of their high frequency of occurrence but may also be important drivers for effecting tumorigenesis in these tumors. Mutations of H3.3 were also observed at low frequency in osteosarcoma (2 % containing G34R in *H3F3A* or *H3F3B*), conventional chondrosarcoma (1 % containing K36M in *H3F3A*), and clear cell chondrosarcoma (7 % containing K36M in *H3F3B*) (Behjati et al. 2013). Interestingly, although highly prevalent in pediatric glioblastoma, to date, no K27M mutations have been observed in bone or cartilage tumors, and the K36M mutant has not been found in glioblastoma.

## A dominant role of the K27M mutation and links with the Polycomb pathway

**Fig. 4:**
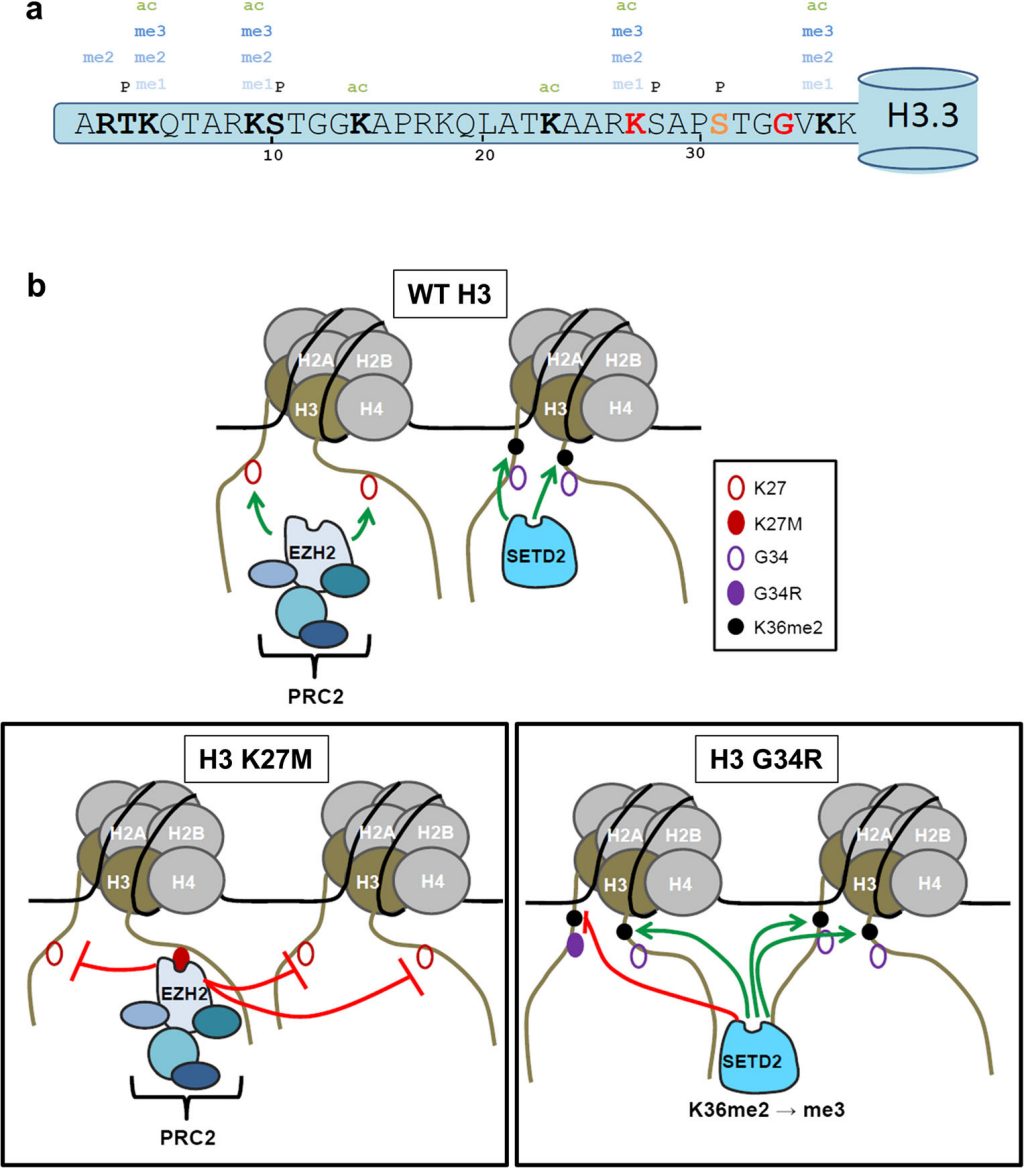
K27M mutants dominantly block PRC2 methyltransferase activity on H3K27, whereas G34R/V mutants block SETD2 methyltransferase function on K36 of the same tail. **a** Representation of the amino terminal tail of histone H3.3 showing the position of known posttranslational modifications, the site of amino acid substitutions identified in tumors (K27 and G34: *red*), and the amino acid that differs in the H3.3 tail from H3.1 (Ser 31: *orange*). **b** Cartoon depicting the distinct modes of action of K27M mutants and G34R/V mutants in modulating posttranslational modifications on H3 proteins. Note that for the K27M mutant, we depict EZH2 as bound to mutant chromatin, with methyltransferase activity blocked on adjacent sites. Such binding of EZH2 ON chromatin may block the chromatin template from additional chromatin transactions. An alternative possibility is that non-nucleosomal K27M mutant H3 could sequester EZH2 off chromatin, which would leave open the possibility of additional modifications occurring on the mutant chromatin template

The N-terminal tails of histones are decorated in covalent posttranslational modifications which can modulate the accessibility of the underlying DNA for diverse chromatin transactions such as transcriptional control, chromosome segregation, and the repair of DNA damage. Modifications on the tail of histone H3 are well documented (Fig. 4a), and the K27M missense mutation has generated much interest due to studies which suggest it plays a dominant role in blocking the accumulation of repressive H3K27 methyl marks (Bender et al. 2013; Lewis et al. 2013; Venneti et al. 2013; Chan et al. 2013). Genomic H3K27 methylation is regulated by the Polycomb group (PcG) of proteins. PcG are evolutionarily conserved proteins which have roles ranging from seed development in plants, X-chromosome inactivation in mammals to maintenance of identity in stem cells. These complexes epigenetically regulate transcriptional states by modulating the H3K27Me3 and H2AK119Ub1 histone marks. In *Drosophila*, PcG proteins interact with PREs to establish cellular memory modules and are involved in developmental determination of body plan by repressing homeotic genes (reviewed in (Di and Helin 2013; Grossniklaus and Paro 2014)). While PRE-like elements in mammals have been reported (Sing et al. 2009; Woo et al. 2010; Cuddapah et al. 2012; Bengani et al. 2013; Woo et al. 2013), their nature and functional relevance are subject to debate. H3K27 is an important target of PRC2 (Polycomb repressive complex 2) complexes that contain EZH2 or EZH1 histone methyl transferases. Indeed in *Drosophila*, K27 has been demonstrated to be a critical target for PRC2 since replacement of the histone H3 cluster with an H3K27R transgene mimicked the phenotype of loss of PRC2 activity (Pengelly et al. 2013), even in the context of a wild-type H3.3 genetic background.

Detection of high-frequency K27M mutations in pediatric DIPG directed examination of the obvious link between H3K27- and PRC2-mediated epigenetic modifications. K27M mutation is predominantly found in H3.3, while ∼20 % of K27M mutations are found in H3.1 (Wu et al. 2012; Saratsis et al. 2014). Most intriguingly, many studies have shown a dominant effect of the K27M mutation, irrespective of whether found in H3.1 or H3.3, with a pronounced reduction in total H3K27 Me2 and Me3 in cells expressing one H3K27M mutant allele among 30 alleles encoding H3 isoforms (Lewis et al. 2013; Chan et al. 2013; Bender et al. 2013; Venneti et al. 2013). In vitro, a H3K27M peptide suffices to block PRC2 activity (Lewis et al. 2013; Brown et al. 2014). The most likely explanation is that K27M “poisons” PRC2, by stabilizing binding of the enzyme to K27M and thus prevents deposition of methyl marks on other H3 proteins (Lewis et al. 2013; Lewis and Allis 2013) (Fig. 4b). In support of this view, K4M mutants in H3 were previously used to mimic H3K4me2 and to stabilize binding of LSD2 to H3 for crystallization studies (Zhang et al. 2013). The level of inhibition of PRC2 activity by K27M is similar to a known chemical EZH2 inhibitor, GSK343 (Bender et al. 2013). Immunoprecipitation of K27M co-purified EZH2 (Chan et al. 2013) and the use of a photoreactive K27M containing peptide to identify binding partners cross-linked primarily the EZH2 subunit of the PRC2 complex (Lewis et al. 2013). Substitution of K27 with methionine and to a lesser extent isoleucine seems sufficient to block the SET catalytic domain of EZH2 by affecting substrate binding and turnover. A similar reduction in SET domain activity was seen for K9M and K36M, but interestingly, not K4M mutants, where lysines are targeted by distinct SET domain containing methyltransferases (Lewis et al. 2013; Herz et al. 2014). An interesting question is whether the stabilized binding of EZH2 to K27M can occur on soluble histone H3 prior to its incorporation into nucleosomes, as suggested by in vitro binding studies to peptides (Brown et al. 2014). If so, only EZH2 function would be blocked, whereas if EZH2 is bound to mutant chromatin, the activity of other nucleosome modifiers or chromatin remodelers may also be affected.

Mis-regulation of PRC2 target genes and mutations predicted to increase or reduce the activity of PRC2 components have been reported in many cancers (Hock 2012), but PRC2 component mutations per se have not been found in glioblastoma. Mutation of p53 in conjunction with expression of H3K27M in nestin expressing progenitor cells of the neonatal brainstem was not sufficient to induce glioma but did induce ectopic cell clusters in the majority of mice that stained positive for Ki-67, which marks proliferating cells (Lewis et al. 2013). The apparent induction of proliferation by expression of K27M appears to be very cell type and developmental stage-specific as K27M did not induce proliferation in undifferentiated human ES cells or in primary human astrocytes (Funato et al. 2014) and indeed suppressed the proliferation of immortalized normal human astrocytes (Buczkowicz et al. 2014), suggesting that there is no driver effect on tumorigenesis of the K27M mutation alone in this cell type. Sequencing of pediatric glioblastomas has revealed the presence of several other coexisting driver mutations in signaling pathway components and in other chromatin regulators in distinct classes of pediatric high-grade gliomas (reviewed in (Jones and Baker 2014)). Generation of mouse models will allow dissection of the interplay and contributions of histone H3 mutants and other mutations for gliomagenesis.

At sites of high transcriptional activity, incorporation of H3.3 is increased relative to H3.1 and H3.2, so in cells expressing H3.3 K27M, this may contribute to enhanced enrichment of the mutant protein in transcriptionally active domains. This may lead to a more potent effect of mutant H3.3 compared to mutant H3.1 in transcriptional dysregulation and contribute to tumorigenesis. In spite of the pronounced reduction in K27 methylation in gliomas expressing low levels of K27M, ChIP-SEQ experiments have revealed that some genomic loci escape this effect and can accumulate high levels of K27me2/3 marks (Chan et al. 2013; Bender et al. 2013). This enrichment for K27 methylation is associated with gene silencing, and genes in this group include cancer-associated genes such as *p16INK4A* and *CDK6*. Genes that were reduced for H3K27me3 marks and were transcriptionally upregulated include the glioma-promoting candidate neural restricted transcription factor *OLIG2* (Chan et al. 2013), which may promote collapse of p53 signaling (Mehta et al. 2011). Additional modifications like reduction in DNA methylation on oncogenic regions of the genome (Bender et al. 2013) may help stabilize the tumor phenotype. How these islands of PRC2 activity are maintained is clearly an interesting question and may be linked to disparate modes of PRC2 recruitment to different loci or to the use of alternate methyltransferases since to date there is no direct demonstration that EZH1 activity is similarly blocked by K27M. Loss of H3K27me3 in K27M expressing cells may also allow for an increase in H3K27Ac (Lewis et al. 2013). The presence of H3K27Ac distinguishes active enhancers (Calo and Wysocka 2013; Vermunt et al. 2014); thus, an aberrant transcriptional program may be elicited through turning on of normally silent enhancers in K27M expressing cells, contributing to tumorigenesis.

Therapeutic attempts by targeting methylation pathways have yielded encouraging results. GSKJ4, a pharmacological JMJD3 (a H3K27me3 demethylase) inhibitor, leads to restoration of H3K27me3, leading to tumor cell lethality in vitro and a significant improvement in the survival of mice that carry tumors (Hashizume et al. 2014). A study by Brown et al. calls for alternative approaches by targeting different histone modification pathways to alter other posttranslational modifications on histones such as H3S28 phosphorylation which can minimize the dominant negative effect of H3K27M (Brown et al. 2014). Hitherto, a suitable model to study the disease was a limiting factor. Recently it was found that co-expression of K27M in the presence of other “driver” mutations (a constitutively active form of platelet-derived growth factor A and loss of p53) in neural progenitor cells derived from human embryonic stem cells promotes neoplastic transformation and induction of low-grade DIPG (Funato et al. 2014). Chemical screens on these induced DIPG cells have identified the menin inhibitor MI2 as a potential drug candidate (Funato et al. 2014). It is interesting to note that menin is a member of the trithorax histone methyltransferase complex and is involved in transcription regulation. Therefore, independent studies across models seem to point toward epigenetic pathways as potential therapeutic targets in treatment of pediatric HGGs. However, the complexity of the genomic landscape of these tumors argues for the importance of performing biopsies to allow for better classification of individual tumors, and that targeting multiple driver mutations may be necessary to achieve therapeutic benefit.

## Disruption of the Set2/K36me3 axis

Lys 36 of histone H3 can undergo mono-, di-, and tri-methylation as well as antagonistic acetylation at the same residue. Posttranslational modification of H3K36 is associated with active transcription, alternative splicing, dosage compensation, DNA replication, and DNA damage repair (Wagner and Carpenter 2012). In yeast, SET domain-containing 2 (Set2) writes all three methylation states at H3K36 whereas in mammals, each state of methylation is laid down by distinct enzymes suggesting extensive regulation of K36 methylation and its importance throughout evolution (Morris et al. 2005; Wagner and Carpenter 2012). Enzymes that modify K36 have been associated with various cancers. For example, *SETD2* (the only enzyme that performs K36me2 to me3 modification) is mutated in renal carcinoma and breast cancer (Duns et al. 2010; Newbold and Mokbel 2010), and *NSD2*, which generates K36me2, has been shown to be a tumor suppressor (Kuo et al. 2011). Recently, missense or truncating mutations in *SETD2* have been reported in pediatric high-grade gliomas of the cerebral hemispheres that do not harbor H3.3 mutations (Fontebasso et al. 2013). It is presently unclear exactly how SETD2 functions as a tumor suppressor, but loss of function may result in an impaired chromatin template for processes such as transcription and DNA repair.

Other tumors mimic loss of SETD2 function by mutation of K36 of H3.3 (to K36M) or by introduction of a mutation in a residue close to K36 (at G34) that may influence binding of writer or reader proteins at K36 (Schwartzentruber et al. 2012; Behjati et al. 2013; Lewis et al. 2013; Chan et al. 2013). Why G34 is targeted, but not other neighboring residues of K36 remains a mystery. It is conceivable that the introduction of a charged (R) or a bulky residue (W) in place of G34 might impact the accessibility or activity of enzymes that target K36 or alter the conformation of the tail leading to changes in nucleosomal packaging that affect the binding of histone readers to H3K36me3. Accordingly, nucleosomes harboring either a G34R or G34V mutant H3.3 exhibit reduced H3K36me2/me3 levels on the same tail, but have no dominant effect on total cellular H3K36me2/me3 levels (in contrast to the reduced K27me3 in H3K27M mutants) (Bjerke et al. 2013; Lewis et al. 2013). It is intriguing that K36 to M and G34W/L mutations have been identified in H3.3 in chondroblastoma and giant cell tumors of bone (Behjati et al. 2013), raising the important question of why the K36 methylation axis is targeted in cancers arising in developmentally distinct tissues. Also, it is important to note that the mutations impacting K36 methylation (K36M and G34 mutants) are only in H3.3, with little rationale for this selection based on codon usage. The selection for these mutations in H3.3 may be explained by the enhanced deposition of H3.3 over H3.1 in transcriptionally active domains and regulatory regions. Deposition of mutant H3.3 would profoundly impact the transcriptional program since loss of K36 methylation negatively impacts transcriptional elongation (Yoh et al. 2008; Carvalho et al. 2013).

The most extensive analysis of the role of G34 mutants has been performed in a cell line generated from a pediatric glioblastoma harboring a G34V mutation in *H3F3A*. The G34V mutation was linked to an altered transcriptional status of the cells, with quite widespread changes in RNA polymerase II association and levels of K36 methylation when compared with a glioma cell line wild-type for H3.3. One locus that was particularly induced was *MYCN*, an oncogene implicated in pediatric glioblastoma (Swartling et al. 2012). Transduction of G34V mutant H3.3 into normal human astrocytes or into transformed human fetal glial cells was sufficient to induce N-MYC expression two- to threefold over cells transduced with WT H3.3 (Bjerke et al. 2013). Such reprogramming of transcription of oncogenes may be critical for tumorigenesis in G34 mutant tumors.

Although global levels of K36 methylation appear unaffected, this mark is reduced on nucleosomes bearing G34R/V mutations, and there clearly are widespread changes in K36 methylation profile of the G34V glioma cell line analyzed (Bjerke et al. 2013) (which may be indirectly caused by the altered transcriptional program of these cells). One possibility is that an altered local H3K36 methylation state may modulate the function of proteins that normally “read” the mark. One interesting example is ZMYND11, a tumor suppressor protein which specifically reads H3K36me3 on the replacement histone H3.3 (Guo et al. 2014; Wen et al. 2014c). G34V/R mutations compromise ZMYND11 binding to the H3.3K36me3 peptide (Wen et al. 2014c); however, a role for ZMYND11 has not been shown to date in any of the pediatric glioblastoma models or in chondroblastoma or giant cell tumors of bone. It is likely that ZMYND11 will be delocalized in chondroblastoma bearing H3.3 K36M mutation, and its localization will be altered in other G34 mutant-expressing tumors where K36 methylation is locally reduced. It will be interesting to determine whether mutation of ZMYND11 or SETD2 occurs in these tumor types and if knockdown of these factors in the appropriate cell types recapitulates features of the diseases.

A second possibility is based on the role of H3K36 in genome stability. Pediatric HGG is characterized by abundant somatic coding mutations, suggestive of defects in DNA damage repair (Jones and Baker 2014). Indeed, microsatellite instability (MSI) is very high in pediatric HGG (Viana-Pereira et al. 2011). H3.3 histones have been shown to be deposited in UVC-damaged regions to restore transcription (Adam et al. 2013), and chicken bursal lymphoma DT40 cells either depleted for histone H3.3 or harboring H3.3 with G34R/V mutation are sensitive to UV (Frey et al. 2014). This is not surprising since H3K36 methylation and SETD2 are involved in DNA damage repair (Carvalho et al. 2014; Jha and Strahl 2014; Pai et al. 2014; Pfister et al. 2014). H3K36me3 may promote DNA repair pathways such as mismatch repair since H3K36me3 recruits the mismatch recognition complex hMutSα onto chromatin through association of the hMSH6 PWWP (Pro-Trp-Trp-Pro) domain with H3K36me3 (Li et al. 2013). H3K36 modification (methylation or acetylation) may also impact the choice of DNA repair pathway between nonhomologous end-joining (NHEJ) and homologous recombination (HR) (Pai et al. 2014), and H3K36 methylation status has also been implicated in determining the timing of origin activity during DNA replication (Pryde et al. 2009). It is interesting that H3.3 is the predominant H3 used for chromatin repair in many cell types (Adam et al. 2014), and that G34 and K36 mutations are exclusively in H3.3. Further exploration of DNA damage pathways in pediatric HGG, chondroblastoma, and giant cell tumors of bone should prove fruitful.

## Conclusions and future work

The identification of high-frequency histone mutations in pediatric high-grade gliomas has given us an inroad to better understand and to treat these deadly tumors. Of particular interest is to determine how K27M mutation in a single copy of histone H3 can have a dominant effect on global H3K27 methylation and, in particular, how some genes escape these effects and accumulate K27me3. Is the mechanism of H3K27M blockage of EZH2 activity restricted to EZH2 or does it impact EZH1 also? What about for K36M mutants in chondroblastoma—are SETD2 or other K36 methyltransferases similarly dominantly blocked for function? It is also critical to determine the mechanism by which G34 mutations influence SETD2 function and how they and the K36M mutation contribute to tumorigenesis.

Why are the mutations found in these tumors so predominant in H3.3? Is it linked to the nature of the replacement histone that can be incorporated into actively transcribed and regulatory regions in nonproliferating cells? Or is it through H3.3 incorporation into repetitive heterochromatic regions and linked to disruption of the genomic stability normally contributed by these specialized domains? Intriguingly, pediatric HGG mutant tumors are sometimes also mutated for ATRX and or DAXX (Khuong-Quang et al. 2012; Schwartzentruber et al. 2012), which incorporate H3.3 into telomeres (Drane et al. 2010; Goldberg et al. 2010). Loss of ATRX/DAXX has been linked to lengthening of telomeres by a telomerase-independent process termed ALT for alternate lengthening of telomeres (Heaphy et al. 2011). ALT is associated with extensive genome rearrangements and defects in double-strand break repair (Lovejoy et al. 2012), reviewed in (O’Sullivan and Almouzni 2014). Pediatric HGG bearing ATRX or DAXX mutations and mutant for H3.3 are characterized by ALT (Schwartzentruber et al. 2012). It will be interesting to determine whether ALT is a feature of histone mutant tumors that are wild-type for ATRX/DAXX, and whether skeletal tumors bearing distinct H3 mutations similarly display association with mutant ATRX/DAXX and ALT.

H3.3 is frequently associated with regions of transcriptional activity. In vitro, there is little difference in stability of nucleosomes bearing H3.3 in place of H3.1, but in vivo, H3.3 is frequently incorporated along with another histone variant, H2AZ, and these nucleosomes are inherently unstable (Jin and Felsenfeld 2007; Chen et al. 2013), which may accentuate transcriptional output of H3.3 domains. H3.3 has been documented to be important for determining chromatin plasticity of developmentally regulated genes in pluripotent mouse embryonic stem cells (Banaszynski et al. 2013). The histone mutant tumors are likely caused by a defect in differentiation during development, and the presence of mutant H3.3 or aberrantly modified H3 proteins (due to dominant effects of K27M or local effects of G34 mutant H3.3) at key regulatory elements may be critical to alteration of transcriptional programs leading to tumor initiation or progression. H3.3 is incorporated into promoters of developmentally regulated genes, and these loci bear both H3K27me3 and H3K4me3 marks, marking these promoters as “bivalent domains” poised for transcriptional induction (Bernstein et al. 2006; Goldberg et al. 2010; Banaszynski et al. 2013). H3.3 is required for establishment of the H3K27me3 mark in these domains (Banaszynski et al. 2013), and it is easy to envisage that a switch to K27Ac or possibly K36me3 may tip the balance to activation of aberrant developmental programs contributing to tumorigenesis.

Much progress has been made in our understanding of these histone mutants since their discovery, but there remains much to be done to develop therapies for pediatric HGG and for the skeletal and bone tumors. We anticipate that the development of additional model systems to interrogate the function of mutant H3 proteins together with insight from analysis of tumors will fuel the collaborative synergy between experts in cancer biology, chromatin biologists, and chemical biologists to develop effective therapies for patients and to improve our understanding of the fascinating biology of histone H3.

## References

[CR1] Adam S, Polo SE, Almouzni G (2013). Transcription recovery after DNA damage requires chromatin priming by the H3.3 histone chaperone HIRA. Cell.

[CR2] Adam S, Polo SE, Almouzni G (2014). How to restore chromatin structure and function in response to DNA damage—let the chaperones play: delivered on 9 July 2013 at the 38th FEBS Congress in St Petersburg, Russia. FEBS J.

[CR3] Ahmad K, Henikoff S (2002). The histone variant H3.3 marks active chromatin by replication-independent nucleosome assembly. Mol Cell.

[CR4] Banaszynski LA, Wen D, Dewell S, Whitcomb SJ, Lin M, Diaz N, Elsasser SJ, Chapgier A, Goldberg AD, Canaani E, Rafii S, Zheng D, Allis CD (2013). Hira-dependent histone H3.3 deposition facilitates PRC2 recruitment at developmental loci in ES cells. Cell.

[CR5] Bechet D, Gielen GG, Korshunov A, Pfister SM, Rousso C, Faury D, Fiset PO, Benlimane N, Lewis PW, Lu C, David AC, Kieran MW, Ligon KL, Pietsch T, Ellezam B, Albrecht S, Jabado N (2014). Specific detection of methionine 27 mutation in histone 3 variants (H3K27M) in fixed tissue from high-grade astrocytomas. Acta Neuropathol.

[CR6] Behjati S, Tarpey PS, Presneau N, Scheipl S, Pillay N, Van LP, Wedge DC, Cooke SL, Gundem G, Davies H, Nik-Zainal S, Martin S, McLaren S, Goodie V, Robinson B, Butler A, Teague JW, Halai D, Khatri B, Myklebost O, Baumhoer D, Jundt G, Hamoudi R, Tirabosco R, Amary MF, Futreal PA, Stratton MR, Campbell PJ, Flanagan AM (2013). Distinct H3F3A and H3F3B driver mutations define chondroblastoma and giant cell tumor of bone. Nat Genet.

[CR7] Bender S, Tang Y, Lindroth AM, Hovestadt V, Jones DT, Kool M, Zapatka M, Northcott PA, Sturm D, Wang W, Radlwimmer B, Hojfeldt JW, Truffaux N, Castel D, Schubert S, Ryzhova M, Seker-Cin H, Gronych J, Johann PD, Stark S, Meyer J, Milde T, Schuhmann M, Ebinger M, Monoranu CM, Ponnuswami A, Chen S, Jones C, Witt O, Collins VP, Von DA, Jabado N, Puget S, Grill J, Helin K, Korshunov A, Lichter P, Monje M, Plass C, Cho YJ, Pfister SM (2013). Reduced H3K27me3 and DNA hypomethylation are major drivers of gene expression in K27M mutant pediatric high-grade gliomas. Cancer Cell.

[CR8] Bengani H, Mendiratta S, Maini J, Vasanthi D, Sultana H, Ghasemi M, Ahluwalia J, Ramachandran S, Mishra RK, Brahmachari V (2013). Identification and validation of a putative polycomb responsive element in the human genome. PLoS ONE.

[CR9] Bernstein BE, Mikkelsen TS, Xie X, Kamal M, Huebert DJ, Cuff J, Fry B, Meissner A, Wernig M, Plath K, Jaenisch R, Wagschal A, Feil R, Schreiber SL, Lander ES (2006). A bivalent chromatin structure marks key developmental genes in embryonic stem cells. Cell.

[CR10] Bjerke L, Mackay A, Nandhabalan M, Burford A, Jury A, Popov S, Bax DA, Carvalho D, Taylor KR, Vinci M, Bajrami I, McGonnell IM, Lord CJ, Reis RM, Hargrave D, Ashworth A, Workman P, Jones C (2013) Histone H3.3 mutations drive pediatric glioblastoma through upregulation of MYCN. Cancer Discov 10.1158/2159-8290.CD-12-0426 PMC3763966 23539269

[CR11] Brown ZZ, Muller MM, Jain SU, Allis CD, Lewis PW, Muir TW (2014). Strategy for “detoxification” of a cancer-derived histone mutant based on mapping its interaction with the methyltransferase PRC2. J Am Chem Soc.

[CR12] Buczkowicz P, Hoeman C, Rakopoulos P, Pajovic S, Letourneau L, Dzamba M, Morrison A, Lewis P, Bouffet E, Bartels U, Zuccaro J, Agnihotri S, Ryall S, Barszczyk M, Chornenkyy Y, Bourgey M, Bourque G, Montpetit A, Cordero F, Castelo-Branco P, Mangerel J, Tabori U, Ho KC, Huang A, Taylor KR, Mackay A, Bendel AE, Nazarian J, Fangusaro JR, Karajannis MA, Zagzag D, Foreman NK, Donson A, Hegert JV, Smith A, Chan J, Lafay-Cousin L, Dunn S, Hukin J, Dunham C, Scheinemann K, Michaud J, Zelcer S, Ramsay D, Cain J, Brennan C, Souweidane MM, Jones C, Allis CD, Brudno M, Becher O, Hawkins C (2014). Genomic analysis of diffuse intrinsic pontine gliomas identifies three molecular subgroups and recurrent activating ACVR1 mutations. Nat Genet.

[CR13] Calo E, Wysocka J (2013). Modification of enhancer chromatin: what, how, and why?. Mol Cell.

[CR14] Carvalho S, Raposo AC, Martins FB, Grosso AR, Sridhara SC, Rino J, Carmo-Fonseca M, de Almeida SF (2013). Histone methyltransferase SETD2 coordinates FACT recruitment with nucleosome dynamics during transcription. Nucleic Acids Res.

[CR15] Carvalho S, Vitor AC, Sridhara SC, Martins FB, Raposo AC, Desterro JM, Ferreira J, de Almeida SF (2014). SETD2 is required for DNA double-strand break repair and activation of the p53-mediated checkpoint. Elife.

[CR16] Chan KM, Fang D, Gan H, Hashizume R, Yu C, Schroeder M, Gupta N, Mueller S, James CD, Jenkins R, Sarkaria J, Zhang Z (2013). The histone H3.3K27M mutation in pediatric glioma reprograms H3K27 methylation and gene expression. Genes Dev.

[CR17] Chen P, Zhao J, Wang Y, Wang M, Long H, Liang D, Huang L, Wen Z, Li W, Li X, Feng H, Zhao H, Zhu P, Li M, Wang QF, Li G (2013). H3.3 actively marks enhancers and primes gene transcription via opening higher-ordered chromatin. Genes Dev.

[CR18] Chow CM, Georgiou A, Szutorisz H, Silva ME, Pombo A, Barahona I, Dargelos E, Canzonetta C, Dillon N (2005). Variant histone H3.3 marks promoters of transcriptionally active genes during mammalian cell division. EMBO Rep.

[CR19] Cuddapah S, Roh TY, Cui K, Jose CC, Fuller MT, Zhao K, Chen X (2012). A novel human polycomb binding site acts as a functional polycomb response element in Drosophila. PLoS ONE.

[CR20] Di CL, Helin K (2013). Transcriptional regulation by Polycomb group proteins. Nat Struct Mol Biol.

[CR21] Drane P, Ouararhni K, Depaux A, Shuaib M, Hamiche A (2010). The death-associated protein DAXX is a novel histone chaperone involved in the replication-independent deposition of H3.3. Genes Dev.

[CR22] Duarte LF, Young AR, Wang Z, Wu HA, Panda T, Kou Y, Kapoor A, Hasson D, Mills NR, Ma’ayan A, Narita M, Bernstein E (2014). Histone H3.3 and its proteolytically processed form drive a cellular senescence programme. Nat Commun.

[CR23] Duns G, van den Berg E, Van DI, Osinga J, Hollema H, Hofstra RM, Kok K (2010). Histone methyltransferase gene SETD2 is a novel tumor suppressor gene in clear cell renal cell carcinoma. Cancer Res.

[CR24] Elsasser SJ, Huang H, Lewis PW, Chin JW, Allis CD, Patel DJ (2012). DAXX envelops a histone H3.3-H4 dimer for H3.3-specific recognition. Nature.

[CR25] Filipescu D, Szenker E, Almouzni G (2013). Developmental roles of histone H3 variants and their chaperones. Trends Genet.

[CR26] Fontebasso AM, Schwartzentruber J, Khuong-Quang DA, Liu XY, Sturm D, Korshunov A, Jones DT, Witt H, Kool M, Albrecht S, Fleming A, Hadjadj D, Busche S, Lepage P, Montpetit A, Staffa A, Gerges N, Zakrzewska M, Zakrzewski K, Liberski PP, Hauser P, Garami M, Klekner A, Bognar L, Zadeh G, Faury D, Pfister SM, Jabado N, Majewski J (2013). Mutations in SETD2 and genes affecting histone H3K36 methylation target hemispheric high-grade gliomas. Acta Neuropathol.

[CR27] Fontebasso AM, Papillon-Cavanagh S, Schwartzentruber J, Nikbakht H, Gerges N, Fiset PO, Bechet D, Faury D, De JN, Ramkissoon LA, Corcoran A, Jones DT, Sturm D, Johann P, Tomita T, Goldman S, Nagib M, Bendel A, Goumnerova L, Bowers DC, Leonard JR, Rubin JB, Alden T, Browd S, Geyer JR, Leary S, Jallo G, Cohen K, Gupta N, Prados MD, Carret AS, Ellezam B, Crevier L, Klekner A, Bognar L, Hauser P, Garami M, Myseros J, Dong Z, Siegel PM, Malkin H, Ligon AH, Albrecht S, Pfister SM, Ligon KL, Majewski J, Jabado N, Kieran MW (2014). Recurrent somatic mutations in ACVR1 in pediatric midline high-grade astrocytoma. Nat Genet.

[CR28] Frank D, Doenecke D, Albig W (2003). Differential expression of human replacement and cell cycle dependent H3 histone genes. Gene.

[CR29] Franklin SG, Zweidler A (1977). Non-allelic variants of histones 2a, 2b and 3 in mammals. Nature.

[CR30] Frey A, Listovsky T, Guilbaud G, Sarkies P, Sale JE (2014). Histone H3.3 is required to maintain replication fork progression after UV damage. Curr Biol.

[CR31] Funato K, Major T, Lewis PW, Allis CD, Tabar V (2014). Use of human embryonic stem cells to model pediatric gliomas with H3.3K27M histone mutation. Science.

[CR32] Gabrielli F, Aden DP, Carrel SC, Von BC, Rane A, Angeletti CA, Hancock R (1984). Histone complements of human tissues, carcinomas, and carcinoma-derived cell lines. Mol Cell Biochem.

[CR33] Gaillard PH, Martini EM, Kaufman PD, Stillman B, Moustacchi E, Almouzni G (1996). Chromatin assembly coupled to DNA repair: a new role for chromatin assembly factor I. Cell.

[CR34] Gielen GH, Gessi M, Hammes J, Kramm CM, Waha A, Pietsch T (2013). H3F3A K27M mutation in pediatric CNS tumors: a marker for diffuse high-grade astrocytomas. Am J Clin Pathol.

[CR35] Goldberg AD, Banaszynski LA, Noh KM, Lewis PW, Elsaesser SJ, Stadler S, Dewell S, Law M, Guo X, Li X, Wen D, Chapgier A, DeKelver RC, Miller JC, Lee YL, Boydston EA, Holmes MC, Gregory PD, Greally JM, Rafii S, Yang C, Scambler PJ, Garrick D, Gibbons RJ, Higgs DR, Cristea IM, Urnov FD, Zheng D, Allis CD (2010). Distinct factors control histone variant H3.3 localization at specific genomic regions. Cell.

[CR36] Grossniklaus U, Paro R (2014) Transcriptional silencing by Polycomb-group proteins. Cold Spring Harb Perspect Biol 6 10.1101/cshperspect.a019331 PMC4413232 25367972

[CR37] Guo R, Zheng L, Park JW, Lv R, Chen H, Jiao F, Xu W, Mu S, Wen H, Qiu J, Wang Z, Yang P, Wu F, Hui J, Fu X, Shi X, Shi YG, Xing Y, Lan F, Shi Y (2014). BS69/ZMYND11 reads and connects histone H3.3 lysine 36 trimethylation-decorated chromatin to regulated Pre-mRNA processing. Mol Cell.

[CR38] Hake SB, Garcia BA, Duncan EM, Kauer M, Dellaire G, Shabanowitz J, Bazett-Jones DP, Allis CD, Hunt DF (2006). Expression patterns and post-translational modifications associated with mammalian histone H3 variants. J Biol Chem.

[CR39] Hashizume R, Andor N, Ihara Y, Lerner R, Gan H, Chen X, Fang D, Huang X, Tom MW, Ngo V, Solomon D, Mueller S, Paris PL, Zhang Z, Petritsch C, Gupta N, Waldman TA, James CD (2014). Pharmacologic inhibition of histone demethylation as a therapy for pediatric brainstem glioma. Nat Med.

[CR40] Heaphy CM, de Wilde RF, Jiao Y, Klein AP, Edil BH, Shi C, Bettegowda C, Rodriguez FJ, Eberhart CG, Hebbar S, Offerhaus GJ, McLendon R, Rasheed BA, He Y, Yan H, Bigner DD, Oba-Shinjo SM, Marie SK, Riggins GJ, Kinzler KW, Vogelstein B, Hruban RH, Maitra A, Papadopoulos N, Meeker AK (2011). Altered telomeres in tumors with ATRX and DAXX mutations. Science.

[CR41] Herz HM, Morgan M, Gao X, Jackson J, Rickels R, Swanson SK, Florens L, Washburn MP, Eissenberg JC, Shilatifard A (2014). Histone H3 lysine-to-methionine mutants as a paradigm to study chromatin signaling. Science.

[CR42] Hock H (2012). A complex Polycomb issue: the two faces of EZH2 in cancer. Genes Dev.

[CR43] Hodl M, Basler K (2009). Transcription in the absence of histone H3.3. Curr Biol.

[CR44] Huang C, Zhu B (2014). H3.3 turnover: a mechanism to poise chromatin for transcription, or a response to open chromatin?. Bioessays.

[CR45] Huang C, Zhang Z, Xu M, Li Y, Li Z, Ma Y, Cai T, Zhu B (2013). H3.3-H4 tetramer splitting events feature cell-type specific enhancers. PLoS Genet.

[CR46] Huether R, Dong L, Chen X, Wu G, Parker M, Wei L, Ma J, Edmonson MN, Hedlund EK, Rusch MC, Shurtleff SA, Mulder HL, Boggs K, Vadordaria B, Cheng J, Yergeau D, Song G, Becksfort J, Lemmon G, Weber C, Cai Z, Dang J, Walsh M, Gedman AL, Faber Z, Easton J, Gruber T, Kriwacki RW, Partridge JF, Ding L, Wilson RK, Mardis ER, Mullighan CG, Gilbertson RJ, Baker SJ, Zambetti G, Ellison DW, Zhang J, Downing JR (2014). The landscape of somatic mutations in epigenetic regulators across 1,000 paediatric cancer genomes. Nat Commun.

[CR47] Jha DK, Strahl BD (2014). An RNA polymerase II-coupled function for histone H3K36 methylation in checkpoint activation and DSB repair. Nat Commun.

[CR48] Jin C, Felsenfeld G (2006). Distribution of histone H3.3 in hematopoietic cell lineages. Proc Natl Acad Sci U S A.

[CR49] Jin C, Felsenfeld G (2007). Nucleosome stability mediated by histone variants H3.3 and H2A.Z. Genes Dev.

[CR50] Jones C, Baker SJ (2014) Unique genetic and epigenetic mechanisms driving paediatric diffuse high-grade glioma. Nat Rev Cancer 14 10.1038/nrc3811 PMC4747023 25230881

[CR51] Khuong-Quang DA, Buczkowicz P, Rakopoulos P, Liu XY, Fontebasso AM, Bouffet E, Bartels U, Albrecht S, Schwartzentruber J, Letourneau L, Bourgey M, Bourque G, Montpetit A, Bourret G, Lepage P, Fleming A, Lichter P, Kool M, Von DA, Sturm D, Korshunov A, Faury D, Jones DT, Majewski J, Pfister SM, Jabado N, Hawkins C (2012). K27M mutation in histone H3.3 defines clinically and biologically distinct subgroups of pediatric diffuse intrinsic pontine gliomas. Acta Neuropathol.

[CR52] Kraushaar DC, Jin W, Maunakea A, Abraham B, Ha M, Zhao K (2013). Genome-wide incorporation dynamics reveal distinct categories of turnover for the histone variant H3.3. Genome Biol.

[CR53] Kuo AJ, Cheung P, Chen K, Zee BM, Kioi M, Lauring J, Xi Y, Park BH, Shi X, Garcia BA, Li W, Gozani O (2011). NSD2 links dimethylation of histone H3 at lysine 36 to oncogenic programming. Mol Cell.

[CR54] Lewis PW, Allis CD (2013) Poisoning the “histone code” in pediatric gliomagenesis. Cell Cycle 12 10.4161/cc.26356 PMC3885631 24036540

[CR55] Lewis PW, Elsaesser SJ, Noh KM, Stadler SC, Allis CD (2010). Daxx is an H3.3-specific histone chaperone and cooperates with ATRX in replication-independent chromatin assembly at telomeres. Proc Natl Acad Sci U S A.

[CR56] Lewis PW, Muller MM, Koletsky MS, Cordero F, Lin S, Banaszynski LA, Garcia BA, Muir TW, Becher OJ, Allis CD (2013). Inhibition of PRC2 activity by a gain-of-function H3 mutation found in pediatric glioblastoma. Science.

[CR57] Li F, Mao G, Tong D, Huang J, Gu L, Yang W, Li GM (2013). The histone mark H3K36me3 regulates human DNA mismatch repair through its interaction with MutSalpha. Cell.

[CR58] Loppin B, Bonnefoy E, Anselme C, Laurencon A, Karr TL, Couble P (2005). The histone H3.3 chaperone HIRA is essential for chromatin assembly in the male pronucleus. Nature.

[CR59] Lovejoy CA, Li W, Reisenweber S, Thongthip S, Bruno J, De LT, De S, Petrini JH, Sung PA, Jasin M, Rosenbluh J, Zwang Y, Weir BA, Hatton C, Ivanova E, Macconaill L, Hanna M, Hahn WC, Lue NF, Reddel RR, Jiao Y, Kinzler K, Vogelstein B, Papadopoulos N, Meeker AK (2012). Loss of ATRX, genome instability, and an altered DNA damage response are hallmarks of the alternative lengthening of telomeres pathway. PLoS Genet.

[CR60] Loyola A, Bonaldi T, Roche D, Imhof A, Almouzni G (2006). PTMs on H3 variants before chromatin assembly potentiate their final epigenetic state. Mol Cell.

[CR61] McKittrick E, Gafken PR, Ahmad K, Henikoff S (2004). Histone H3.3 is enriched in covalent modifications associated with active chromatin. Proc Natl Acad Sci U S A.

[CR62] Mehta S, Huillard E, Kesari S, Maire CL, Golebiowski D, Harrington EP, Alberta JA, Kane MF, Theisen M, Ligon KL, Rowitch DH, Stiles CD (2011). The central nervous system-restricted transcription factor Olig2 opposes p53 responses to genotoxic damage in neural progenitors and malignant glioma. Cancer Cell.

[CR63] Mito Y, Henikoff JG, Henikoff S (2007). Histone replacement marks the boundaries of cis-regulatory domains. Science.

[CR64] Morris SA, Shibata Y, Noma K, Tsukamoto Y, Warren E, Temple B, Grewal SI, Strahl BD (2005). Histone H3 K36 methylation is associated with transcription elongation in Schizosaccharomyces pombe. Eukaryot Cell.

[CR65] Newbold RF, Mokbel K (2010). Evidence for a tumour suppressor function of SETD2 in human breast cancer: a new hypothesis. Anticancer Res.

[CR66] Osley MA (1991). The regulation of histone synthesis in the cell cycle. Annu Rev Biochem.

[CR67] O’Sullivan RJ, Almouzni G (2014). Assembly of telomeric chromatin to create ALTernative endings. Trends Cell Biol.

[CR68] Pai CC, Deegan RS, Subramanian L, Gal C, Sarkar S, Blaikley EJ, Walker C, Hulme L, Bernhard E, Codlin S, Bahler J, Allshire R, Whitehall S, Humphrey TC (2014). A histone H3K36 chromatin switch coordinates DNA double-strand break repair pathway choice. Nat Commun.

[CR69] Pengelly AR, Copur O, Jackle H, Herzig A, Muller J (2013). A histone mutant reproduces the phenotype caused by loss of histone-modifying factor Polycomb. Science.

[CR70] Pfister SX, Ahrabi S, Zalmas LP, Sarkar S, Aymard F, Bachrati CZ, Helleday T, Legube G, La Thangue NB, Porter AC, Humphrey TC (2014). SETD2-dependent histone H3K36 trimethylation is required for homologous recombination repair and genome stability. Cell Rep.

[CR71] Pina B, Suau P (1987). Changes in histones H2A and H3 variant composition in differentiating and mature rat brain cortical neurons. Dev Biol.

[CR72] Plass C, Pfister SM, Lindroth AM, Bogatyrova O, Claus R, Lichter P (2013). Mutations in regulators of the epigenome and their connections to global chromatin patterns in cancer. Nat Rev Genet.

[CR73] Pryde F, Jain D, Kerr A, Curley R, Mariotti FR, Vogelauer M (2009). H3 k36 methylation helps determine the timing of cdc45 association with replication origins. PLoS ONE.

[CR74] Ray-Gallet D, Woolfe A, Vassias I, Pellentz C, Lacoste N, Puri A, Schultz DC, Pchelintsev NA, Adams PD, Jansen LE, Almouzni G (2011). Dynamics of histone H3 deposition in vivo reveal a nucleosome gap-filling mechanism for H3.3 to maintain chromatin integrity. Mol Cell.

[CR75] Sakai A, Schwartz BE, Goldstein S, Ahmad K (2009). Transcriptional and developmental functions of the H3.3 histone variant in Drosophila. Curr Biol.

[CR76] Santenard A, Ziegler-Birling C, Koch M, Tora L, Bannister AJ, Torres-Padilla ME (2010). Heterochromatin formation in the mouse embryo requires critical residues of the histone variant H3.3. Nat Cell Biol.

[CR77] Saratsis AM, Kambhampati M, Snyder K, Yadavilli S, Devaney JM, Harmon B, Hall J, Raabe EH, An P, Weingart M, Rood BR, Magge SN, MacDonald TJ, Packer RJ, Nazarian J (2014). Comparative multidimensional molecular analyses of pediatric diffuse intrinsic pontine glioma reveals distinct molecular subtypes. Acta Neuropathol.

[CR78] Schwartz BE, Ahmad K (2005). Transcriptional activation triggers deposition and removal of the histone variant H3.3. Genes Dev.

[CR79] Schwartzentruber J, Korshunov A, Liu XY, Jones DT, Pfaff E, Jacob K, Sturm D, Fontebasso AM, Quang DA, Tonjes M, Hovestadt V, Albrecht S, Kool M, Nantel A, Konermann C, Lindroth A, Jager N, Rausch T, Ryzhova M, Korbel JO, Hielscher T, Hauser P, Garami M, Klekner A, Bognar L, Ebinger M, Schuhmann MU, Scheurlen W, Pekrun A, Fruhwald MC, Roggendorf W, Kramm C, Durken M, Atkinson J, Lepage P, Montpetit A, Zakrzewska M, Zakrzewski K, Liberski PP, Dong Z, Siegel P, Kulozik AE, Zapatka M, Guha A, Malkin D, Felsberg J, Reifenberger G, Von DA, Ichimura K, Collins VP, Witt H, Milde T, Witt O, Zhang C, Castelo-Branco P, Lichter P, Faury D, Tabori U, Plass C, Majewski J, Pfister SM, Jabado N (2012). Driver mutations in histone H3.3 and chromatin remodelling genes in paediatric glioblastoma. Nature.

[CR80] Shibahara K, Stillman B (1999). Replication-dependent marking of DNA by PCNA facilitates CAF-1-coupled inheritance of chromatin. Cell.

[CR81] Sing A, Pannell D, Karaiskakis A, Sturgeon K, Djabali M, Ellis J, Lipshitz HD, Cordes SP (2009). A vertebrate Polycomb response element governs segmentation of the posterior hindbrain. Cell.

[CR82] Sturm D, Witt H, Hovestadt V, Khuong-Quang DA, Jones DT, Konermann C, Pfaff E, Tonjes M, Sill M, Bender S, Kool M, Zapatka M, Becker N, Zucknick M, Hielscher T, Liu XY, Fontebasso AM, Ryzhova M, Albrecht S, Jacob K, Wolter M, Ebinger M, Schuhmann MU, Van MT, Fruhwald MC, Hauch H, Pekrun A, Radlwimmer B, Niehues T, Von KG, Durken M, Kulozik AE, Madden J, Donson A, Foreman NK, Drissi R, Fouladi M, Scheurlen W, Von DA, Monoranu C, Roggendorf W, Herold-Mende C, Unterberg A, Kramm CM, Felsberg J, Hartmann C, Wiestler B, Wick W, Milde T, Witt O, Lindroth AM, Schwartzentruber J, Faury D, Fleming A, Zakrzewska M, Liberski PP, Zakrzewski K, Hauser P, Garami M, Klekner A, Bognar L, Morrissy S, Cavalli F, Taylor MD, Van SP, Koster J, Versteeg R, Volckmann R, Mikkelsen T, Aldape K, Reifenberger G, Collins VP, Majewski J, Korshunov A, Lichter P, Plass C, Jabado N, Pfister SM (2012). Hotspot mutations in H3F3A and IDH1 define distinct epigenetic and biological subgroups of glioblastoma. Cancer Cell.

[CR83] Swartling FJ, Savov V, Persson AI, Chen J, Hackett CS, Northcott PA, Grimmer MR, Lau J, Chesler L, Perry A, Phillips JJ, Taylor MD, Weiss WA (2012). Distinct neural stem cell populations give rise to disparate brain tumors in response to N-MYC. Cancer Cell.

[CR84] Tagami H, Ray-Gallet D, Almouzni G, Nakatani Y (2004). Histone H3.1 and H3.3 complexes mediate nucleosome assembly pathways dependent or independent of DNA synthesis. Cell.

[CR85] Taylor KR, Mackay A, Truffaux N, Butterfield YS, Morozova O, Philippe C, Castel D, Grasso CS, Vinci M, Carvalho D, Carcaboso AM, De TC, Cruz O, Mora J, Entz-Werle N, Ingram WJ, Monje M, Hargrave D, Bullock AN, Puget S, Yip S, Jones C, Grill J (2014). Recurrent activating ACVR1 mutations in diffuse intrinsic pontine glioma. Nat Genet.

[CR86] Taylor KR, Vinci M, Bullock AN, Jones C (2014). ACVR1 mutations in DIPG: lessons learned from FOP. Cancer Res.

[CR87] Torres-Padilla ME, Bannister AJ, Hurd PJ, Kouzarides T, Zernicka-Goetz M (2006). Dynamic distribution of the replacement histone variant H3.3 in the mouse oocyte and preimplantation embryos. Int J Dev Biol.

[CR88] van der Heijden GW, Dieker JW, Derijck AA, Muller S, Berden JH, Braat DD, Van DV, De BP (2005). Asymmetry in histone H3 variants and lysine methylation between paternal and maternal chromatin of the early mouse zygote. Mech Dev.

[CR89] Venneti S, Garimella MT, Sullivan LM, Martinez D, Huse JT, Heguy A, Santi M, Thompson CB, Judkins AR (2013). Evaluation of histone 3 lysine 27 trimethylation (H3K27me3) and enhancer of Zest 2 (EZH2) in pediatric glial and glioneuronal tumors shows decreased H3K27me3 in H3F3A K27M mutant glioblastomas. Brain Pathol.

[CR90] Venneti S, Santi M, Felicella MM, Yarilin D, Phillips JJ, Sullivan LM, Martinez D, Perry A, Lewis PW, Thompson CB, Judkins AR (2014). A sensitive and specific histopathologic prognostic marker for H3F3A K27M mutant pediatric glioblastomas. Acta Neuropathol.

[CR91] Vermunt MW, Reinink P, Korving J, De BE, Creyghton PM, Basak O, Geeven G, Toonen PW, Lansu N, Meunier C, Van HS, Clevers H, De LW, Cuppen E, Creyghton MP (2014). Large-scale identification of coregulated enhancer networks in the adult human brain. Cell Rep.

[CR92] Viana-Pereira M, Lee A, Popov S, Bax DA, Al-Sarraj S, Bridges LR, Stavale JN, Hargrave D, Jones C, Reis RM (2011). Microsatellite instability in pediatric high grade glioma is associated with genomic profile and differential target gene inactivation. PLoS ONE.

[CR93] Vogelstein B, Papadopoulos N, Velculescu VE, Zhou S, Diaz LA, Kinzler KW (2013). Cancer genome landscapes. Science.

[CR94] Wagner EJ, Carpenter PB (2012). Understanding the language of Lys36 methylation at histone H3. Nat Rev Mol Cell Biol.

[CR95] Wells D, Kedes L (1985). Structure of a human histone cDNA: evidence that basally expressed histone genes have intervening sequences and encode polyadenylylated mRNAs. Proc Natl Acad Sci U S A.

[CR96] Wells D, Hoffman D, Kedes L (1987). Unusual structure, evolutionary conservation of non-coding sequences and numerous pseudogenes characterize the human H3.3 histone multigene family. Nucleic Acids Res.

[CR97] Wen D, Banaszynski LA, Liu Y, Geng F, Noh KM, Xiang J, Elemento O, Rosenwaks Z, Allis CD, Rafii S (2014). Histone variant H3.3 is an essential maternal factor for oocyte reprogramming. Proc Natl Acad Sci U S A.

[CR98] Wen D, Banaszynski LA, Rosenwaks Z, Allis CD, Rafii S (2014). H3.3 replacement facilitates epigenetic reprogramming of donor nuclei in somatic cell nuclear transfer embryos. Nucleus.

[CR99] Wen H, Li Y, Xi Y, Jiang S, Stratton S, Peng D, Tanaka K, Ren Y, Xia Z, Wu J, Li B, Barton MC, Li W, Li H, Shi X (2014). ZMYND11 links histone H3.3K36me3 to transcription elongation and tumour suppression. Nature.

[CR100] Wong LH, McGhie JD, Sim M, Anderson MA, Ahn S, Hannan RD, George AJ, Morgan KA, Mann JR, Choo KH (2010). ATRX interacts with H3.3 in maintaining telomere structural integrity in pluripotent embryonic stem cells. Genome Res.

[CR101] Woo CJ, Kharchenko PV, Daheron L, Park PJ, Kingston RE (2010). A region of the human HOXD cluster that confers polycomb-group responsiveness. Cell.

[CR102] Woo CJ, Kharchenko PV, Daheron L, Park PJ, Kingston RE (2013). Variable requirements for DNA-binding proteins at polycomb-dependent repressive regions in human HOX clusters. Mol Cell Biol.

[CR103] Workman JL, Kingston RE (1998). Alteration of nucleosome structure as a mechanism of transcriptional regulation. Annu Rev Biochem.

[CR104] Wu RS, Tsai S, Bonner WM (1982). Patterns of histone variant synthesis can distinguish G0 from G1 cells. Cell.

[CR105] Wu G, Broniscer A, McEachron TA, Lu C, Paugh BS, Becksfort J, Qu C, Ding L, Huether R, Parker M, Zhang J, Gajjar A, Dyer MA, Mullighan CG, Gilbertson RJ, Mardis ER, Wilson RK, Downing JR, Ellison DW, Zhang J, Baker SJ (2012). Somatic histone H3 alterations in pediatric diffuse intrinsic pontine gliomas and non-brainstem glioblastomas. Nat Genet.

[CR106] Wu G, Diaz AK, Paugh BS, Rankin SL, Ju B, Li Y, Zhu X, Qu C, Chen X, Zhang J, Easton J, Edmonson M, Ma X, Lu C, Nagahawatte P, Hedlund E, Rusch M, Pounds S, Lin T, Onar-Thomas A, Huether R, Kriwacki R, Parker M, Gupta P, Becksfort J, Wei L, Mulder HL, Boggs K, Vadodaria B, Yergeau D, Russell JC, Ochoa K, Fulton RS, Fulton LL, Jones C, Boop FA, Broniscer A, Wetmore C, Gajjar A, Ding L, Mardis ER, Wilson RK, Taylor MR, Downing JR, Ellison DW, Zhang J, Baker SJ (2014). The genomic landscape of diffuse intrinsic pontine glioma and pediatric non-brainstem high-grade glioma. Nat Genet.

[CR107] Yoh SM, Lucas JS, Jones KA (2008). The Iws1:Spt6:CTD complex controls cotranscriptional mRNA biosynthesis and HYPB/Setd2-mediated histone H3K36 methylation. Genes Dev.

[CR108] Zhang Q, Qi S, Xu M, Yu L, Tao Y, Deng Z, Wu W, Li J, Chen Z, Wong J (2013). Structure-function analysis reveals a novel mechanism for regulation of histone demethylase LSD2/AOF1/KDM1b. Cell Res.

